# Investigating Cortical Responses to Noise-Vocoded Speech in Children with Normal Hearing Using Functional Near-Infrared Spectroscopy (fNIRS)

**DOI:** 10.1007/s10162-021-00817-z

**Published:** 2021-09-28

**Authors:** Faizah Mushtaq, Ian M. Wiggins, Pádraig T. Kitterick, Carly A. Anderson, Douglas E. H. Hartley

**Affiliations:** 1 National Institute for Health Research Nottingham Biomedical Research Centre, Nottingham, NG1 5DU UK; 2 Hearing Sciences, Division of Clinical Neuroscience, School of Medicine, University of Nottingham, Nottingham, NG7 2UH UK; 3 Nottingham University Hospitals NHS Trust, Nottingham, NG7 2UH UK

**Keywords:** temporal cortex, auditory processing, speech perception, neuroimaging, language

## Abstract

Whilst functional neuroimaging has been used to investigate cortical processing of degraded speech in adults, much less is known about how these signals are processed in children. An enhanced understanding of cortical correlates of poor speech perception in children would be highly valuable to oral communication applications, including hearing devices. We utilised vocoded speech stimuli to investigate brain responses to degraded speech in 29 normally hearing children aged 6–12 years. Intelligibility of the speech stimuli was altered in two ways by (i) reducing the number of spectral channels and (ii) reducing the amplitude modulation depth of the signal. A total of five different noise-vocoded conditions (with zero, partial or high intelligibility) were presented in an event-related format whilst participants underwent functional near-infrared spectroscopy (fNIRS) neuroimaging. Participants completed a word recognition task during imaging, as well as a separate behavioural speech perception assessment. fNIRS recordings revealed statistically significant sensitivity to stimulus intelligibility across several brain regions. More intelligible stimuli elicited stronger responses in temporal regions, predominantly within the left hemisphere, while right inferior parietal regions showed an opposite, negative relationship. Although there was some evidence that partially intelligible stimuli elicited the strongest responses in the left inferior frontal cortex, a region previous studies have suggested is associated with effortful listening in adults, this effect did not reach statistical significance. These results further our understanding of cortical mechanisms underlying successful speech perception in children. Furthermore, fNIRS holds promise as a clinical technique to help assess speech intelligibility in paediatric populations.

## **INTRODUCTION**

Noise-vocoding is a signal processing strategy that enables speech signals to be degraded (Dudley, 1939; Shannon et al. 1995). Varying either the number of spectral channels or the amplitude of the modulating envelope of vocoded stimuli can generate speech conditions with differing levels of intelligibility (Newman and Chatterjee, 2013; Lawrence et al. 2018). These stimuli help to advance our understanding of factors underlying speech perception when hearing in impoverished listening situations, or when speech signals are degraded (Roman et al. 2017). Whilst functional neuroimaging has been used to explore cortical representation of degraded speech in adults (Wijayasiri et al. 2017; Lawrence et al. 2018), relatively little is known about how these signals are processed in the brains of children.

Functional near-infrared spectroscopy (fNIRS) is an optically-based technique that is particularly suited to paediatric research. It is quiet, non-invasive, portable, and child-friendly as it is relatively resistant to head movements so babies and children can be scanned whilst awake, and the flexible optic fibres even enable infants to be sat on a parent’s knee during imaging (for a review see Quaresima et al. 2012). Cortical responses in temporal and frontal brain regions to clear speech (e.g., Anderson et al. 2017, 2019) and noise vocoded speech stimuli (e.g., Wijayasiri et al. 2017; Lawrence et al. 2018) have been investigated using fNIRS in adults. A more recent study conducted in our laboratory in school-aged children revealed that clear speech elicited stronger responses in left temporal cortex compared with degraded stimuli (Lawrence et al. 2021). However, this study did not explore the effects of varying both the number of spectral channels and amplitude modulation (AM) depth. The aim of the current study was to examine the effects of speech degradation, using vocoded stimuli of zero, partial, or high intelligibility, on cortical responses measured from temporal and frontal brain regions in children.

Previous psychophysical studies in both adults and children have shown that noise-vocoded stimuli with eight or more spectral channels are highly intelligible (Dorman et al. 1997; Eisenberg et al. 2000). Therefore, we simulated intelligible listening in our sample of children using an eight-channel noise-vocoder. Paediatric data show that it is challenging for children to perceive speech (i.e., identify words correctly) with fewer than four vocoded channels (Eisenberg et al. 2000; Newman and Chatterjee, 2013). Thus, partial intelligibility and unintelligible listening conditions were studied using a (i) four-channel and (ii) single-channel vocoder, respectively. It is important to note that successful performance on different speech tasks is known to require different numbers of channels in different age groups. For example, in studies utilising phoneme discrimination tasks, NH infants aged 6 months discriminated voicing from AM cues extracted from only four frequency bands (Cabrera et al. 2013), whereas more than sixteen channels were necessary in infants of the same age for vowel discrimination (Warner-Czyz et al. 2014). Since our work involved older children and focussed on neuroimaging of sentence-level speech perception, we selected the numbers of channels for each condition accordingly. Furthermore, speech signals are highly modulated and sensitivity to amplitude envelope modulations is essential for successful speech comprehension (Rosen, 1992; Purcell et al. 2004), with reduced AM sensitivity associated with poor speech outcomes in patient groups (e.g., De Ruiter et al. 2015). Therefore, we also temporally degraded speech signals by reducing the AM depth of the stimulus (Lawrence et al. 2018). We hypothesised that more intelligible conditions (i.e., eight spectral channels or full AM depth) would elicit stronger responses in temporal brain regions compared with partially intelligible (i.e., four spectral channels or reduced AM) or unintelligible (i.e., one spectral channel or zero AM) conditions (Lawrence et al. 2018; Cabrera and Gervain, 2020).

Finally, evidence suggests that increased activation in left inferior frontal brain regions is correlated with an increased attentional demand and effortful listening to degraded speech (Wild et al. 2012; Wijayasiri et al. 2017; Lawrence et al. 2018). Therefore, we also investigated cortical correlates of effortful listening in this brain region, hypothesizing greater cortical activity in response to partially intelligible speech than to either unintelligible or highly intelligible speech. Furthermore, the responsiveness of left posterior temporal cortex (“Wernicke’s area”) also correlates with speech intelligibility (e.g., Mottonen et al. 2006; Lawrence et al. 2018), signifying that this area plays an important role in higher-level speech processing (Wise et al. 2001; Hassanpour et al. 2015). Consequently, we investigated the effects of speech intelligibility on cortical responses in this brain region, expecting to observe stronger cortical activity in response to more intelligible stimuli.

## **MATERIALS AND METHODS**

### Participants

Twenty-nine children (mean age 9.8 years; age range 6–12 years; 16 males) volunteered to take part in the study. Participants were mainly recruited via online advertisements. All children were native English speakers with no known hearing problems, normal or corrected-to-normal vision and no history of language, cognitive, or motor impairment. They all scored 100 % on a pure tone audiometry air-conduction hearing screen performed at 20 dB HL at 1, 2, 4, and 0.5 kHz respectively in both ears (procedure adapted from the British Society of Audiology (BSA, 2018)). Non-verbal intelligence was assessed using the block design and matrix reasoning subsets from the Wechsler Abbreviated Scale of Intelligence – Second Edition (WASI-II) (Wechsler, 2011; McCrimmon and Smith, 2012), with the group average age-corrected IQ ranked at the 53rd percentile (range 1st to 96th percentile). A motor-speech laterality questionnaire by Flowers and Hudson (2013) indicated that twenty-four children were right-handed. Written informed consent was obtained from the accompanying parents, guardians, or relatives of all participants, and subjects were also asked to verbally agree to participate. The study was approved by the University of Nottingham Faculty of Medicine and Health Sciences Research Ethics Committee.

### Stimuli

Five stimulus conditions were generated for the study. These were (i) one-channel noise-vocoded speech with full AM depth (1-ch-F), (ii) four-channel noise-vocoded speech with full AM depth (4-ch-F), (iii) eight-channel noise-vocoded speech with full AM depth (8-ch-F) (iv) eight-channel noise-vocoded speech with reduced AM depth (8-ch-R), and (v) eight-channel noise-vocoded speech with zero AM depth (8-ch-Z). The 8-ch-F stimulus was the clearest and most intelligible (“high intelligibility”), the 4-ch-F and 8-ch-R conditions were less clear but still intelligible to some degree (“partial intelligibility”), and the 1-ch-F and 8-ch-Z stimuli were both completely unintelligible (“zero intelligibility”).

Recordings of Bamford-Kowal-Bench (BKB) sentences (Bench et al. 1979) recited by a male speaker were used as auditory stimuli. Three hundred and twenty sentences were available in total. Stimuli were created using scripts previously developed in our laboratory (Wijayasiri et al. 2017; Lawrence et al. 2018). One, four or eight-channel noise-vocoding was applied to the auditory sentences (Shannon et al. 1995). The channels covered the range from 180 to 8000 Hz and were spread approximately equally along the basilar membrane (Greenwood, 1990). The MATLAB (Mathworks, Natick, MA) *filtfilt* function was used to successively apply sixth order digital elliptic filters in the forward and backward directions to prevent phase distortion. Half-wave rectification and zero-phase low-pass filtering using a first order elliptic filter (applied successively in the forward and backward directions) was performed to extract within-channel amplitude envelopes.

For the 8-ch-R condition, the AM depth within each channel was then altered by raising the extracted envelopes to a fractional power (Fu and Shannon, 1998; Shannon, 2002). Based upon power law mapping, the amplitude of the output is equal to the input amplitude raised to a power (Shannon, 2002). Therefore, envelope exponents of less than one result in compression of amplitude and, conversely, exponents greater than one result in expansion of amplitude (Shannon, 2002). In this study, an envelope exponent of 0.37 was used which was consistent across all channels. This manipulation method has been used in previous fNIRS work conducted in our laboratory and an exponent of 0.37 was selected as the resulting signal is of comparable difficulty to four-channel noise-vocoded speech (with full AM) in adults (Lawrence et al. 2018). Therefore, speech intelligibility in both the partially intelligible (8-ch-R and 4-ch-F) listening conditions was expected to be approximately equal. Note that while similar intelligibility was expected, we anticipated that the neural mechanisms involved may differ due to substantial differences in the manipulation of the acoustic parameters (Zatorre et al. 2002).

For the 8-ch-Z condition, an envelope exponent of zero was used (equivalent to completely unintelligible steady speech-shaped noise). For the remaining three conditions (8-ch-F, 4-ch-F, and 1-ch-F), which were all fully modulated, an envelope exponent of one was applied, which is equivalent to the envelope extracted from the original speech. Note that the 1-ch-F condition was also completely unintelligible owing to a lack of spectral resolution.

Following the relevant manipulation of the envelopes appropriate for each condition, each envelope was then applied to a white-noise carrier and bandpass filtered with the same filters used to split the input signal up into either one, four, or eight vocoder channels. The output of each vocoder channel was filtered in this way to ensure that only the relevant part of the frequency spectrum would be excited for each channel. Input and output root-mean-square levels were matched on a within-channel basis, followed by summation across channels. All speech stimuli were processed using MATLAB.

### Experimental Procedure

#### Main Neuroimaging Task

Fourteen different BKB sentences (Bench et al. 1979) were presented at random for each condition, with a further fourteen muted sentences used to form a silent baseline condition (eighty-four BKB sentences in total). The average duration of each sentence was 1.64 s (range 0.86 to 2.30 s). Stimulus onset asynchrony (the time between the onset of one sentence and the next) was varied randomly in the range 6 to 9 s to improve efficiency and enable temporally-overlapping responses to be deconvolved (Dale, 1999). Similar to an adult fNIRS study previously conducted in our laboratory (Lawrence et al. 2018), a probe word appeared centrally on a display screen 0.5 s after the presentation of each auditory stimulus. The probe word was a word that had appeared in the previous sentence or a foil word that rhymed with one of the actual keywords. The probability of either a true keyword or foil word being displayed was equal, as was the probability of the probe word occurring near the beginning, middle, or end of the preceding stimulus. To encourage the subjects to actively attend to the auditory stimuli, they were instructed to listen to the auditory stimuli and press a button on a response box (“RTbox”) (Li et al. 2010) as quickly as possible to indicate whether the probe word displayed had featured in the sentence they had just listened to. Participants were told in advance which two buttons on the response box (one on the left side and one on the right side) corresponded to “Yes” they had heard the probe word or “No” they had not. To assist with this, underneath the probe word, a “Yes” and “No” label was shown on each side of the screen that corresponded with the two relevant buttons. These labels remained consistent throughout each participant’s session but were reversed after each individual so that half of the subjects indicated “Yes” by pressing the right-sided button on the response box and the other half by pressing the left-sided button. For silent trials, the participants were told to follow instructions on the screen that would indicate, at random, whether to “Press yes” or “Press no.” Subjects had up to 3 s to respond, after which a missing response would otherwise be logged and the experiment would move on to the next trial.

Similar to our previous fNIRS work with NH children of comparable age (Mushtaq et al. 2019), participants were able to track their progress by counting stars that were displayed for 4 s as each fifth of the experiment elapsed. This provided subjects with additional encouragement and offered a sense of duration as the task ended after all five stars had been collected. When the reward stars and probe words were not shown, a grey background was displayed on the screen along with a small, centrally positioned fixation cross that participants were instructed to look at throughout.

One run of the main fNIRS imaging task (i.e., consisting of 14 sentences per condition) lasted approximately 11.5 min in total. Twenty-seven participants completed two runs of the fNIRS imaging task with a break in-between, with the remaining two subjects completing one run due to fatigue. All participants completed a short practice session in order to become familiar with the task and stimuli before the fNIRS optode array was positioned on their head. This practice task was repeated more than once if the subject made errors until the investigator was satisfied that the participant understood the task fully.

#### Speech Perception Test and Familiarisation Task

Participants completed a behavioural speech perception test before (pre-imaging) and after (post-imaging) the main fNIRS task to assess their ability to understand the noise-vocoded stimuli. By splitting the speech perception test into two parts, conducted before and after imaging, we assumed that each individual’s speech perception ability during the main fNIRS task would have fallen somewhere in between the two behavioural measurements. Twenty-five participants completed the pre-imaging speech perception test, followed by the main fNIRS task in full (either one or two runs depending on the subject) before ending with the post-imaging speech perception test. Four subjects completed the post-imaging speech perception test after the first run of the main fNIRS task and then proceeded to complete the second run of the main fNIRS task.

During each speech perception test, participants were presented with eight sentences per condition, resulting in forty sentences per test (eighty sentences in total across the pre- and post-imaging tests). The order of presentation was randomised. Subjects were instructed to carefully listen to the sentences and repeat them back to the experimenter to the best of their ability. The experimenter scored each subject’s responses against pre-determined keywords. An example sentence with the keywords underlined is as follows: The bag bumps on the ground. For each condition, there were between twenty-four and twenty-eight keywords for each part of the test.

In order to introduce listening to degraded speech signals to the children and to offer them an opportunity to practise the task, a short familiarisation task preceded the start of the pre-imaging speech perception test. Four sentences were presented per condition, and the task instructions were the same as those for the speech perception test. This familiarisation task was not scored and was performed simply to familiarise subjects with listening to noise-vocoded signals.

Note that no BKB sentence (Bench et al. 1979) was presented twice to any individual participant during the experiment, with different sentences selected at random for both parts of the speech perception test, the initial stimuli familiarisation and fNIRS practice tasks, and the two fNIRS runs.

### Equipment

Cortical activation was measured using a continuous wave fNIRS system (ETG-4000, Hitachi Medical Co., Tokyo, Japan) which minimises crosstalk between channels and wavelengths using frequency modulation (for review see Scholkmann et al. 2014). Responses were measured from 44 channels (22 channels per hemisphere) at wavelengths of 695 nm and 830 nm (sampling rate 10 Hz) using thirty optodes arranged into two 3 × 5 arrays with a 3-cm fixed source-detector gap. The International 10–20 positioning system (Jasper, 1958) was used to guide consistent array placement across participants. Similar to our previous fNIRS work with a comparable age group (Mushtaq et al. 2019), the middle optode on the top row was directed towards point Cz and the middle optode on the bottom row was positioned as close to the preauricular point as possible. To maximise optode-scalp contact, hair was moved from underneath optodes using a small plastic illuminated tool and a photograph taken of the final array position for reference purposes. Testing was carried out within a sound-treated room with dimmed lighting. Participants were comfortably seated approximately 75 cm from a visual display monitor and a Genelec 8030A loudspeaker which presented the auditory stimuli in the free-field at a level of 65 dB SPL (A-weighted root-mean-square level averaged over the duration of each sentence), measured at the participant’s listening position without the participant present using a sound level meter (Type 2250, Brüel & Kjær, Nærum, Denmark). The experiment was programmed in MATLAB using the Psychtoolbox-3 extensions (Brainard, 1997; Pelli, 1997; Kleiner et al. 2007).

### Data Analysis

#### fNIRS Data Analysis

fNIRS analyses were conducted in MATLAB with HOMER2 functions (Huppert et al. 2009) using custom scripts developed in our laboratory and used across multiple previous studies (Wiggins et al. 2016; Anderson et al. 2017, 2019; Wijayasiri et al. 2017; Lawrence et al. 2018, 2021; Mushtaq et al. 2019). Initially, the worst 5 % of channels with the poorest optode-scalp contact were excluded using the scalp coupling index (SCI) method by Pollonini et al. (2014). We used a relatively permissive SCI threshold (≥ 0.07) to exclude the worst channels whilst retaining as many channels as possible for subsequent statistical analysis. The HOMER2 *hmrIntensity2OD function* (Huppert et al. 2009) was used to convert the raw fNIRS light intensity levels into changes in optical density. Motion artefact correction was applied using the HOMER2 *hmrMotionCorrectionWavelet* function (Molavi and Dumont, 2012) with wavelet coefficients lying more than 0.719 times the interquartile range below the first or above the third quartiles removed. Next, cardiac oscillations and low-frequency drift were attenuated by bandpass filtering the data between 0.02 and 0.5 Hz. The modified Beer-Lambert law was applied to convert optical density signals into estimates of oxygenated haemoglobin (HbO) and deoxygenated haemoglobin (HbR) (Huppert et al. 2009). At both wavelengths, a default value of 6 was used for the differential path-length factor. A signal separation algorithm by Yamada et al. (2012) was applied to isolate the functional component of the haemodynamic signal (which was entered into the general linear model described below), the application of which has been shown to improve the reliability of fNIRS responses recorded from temporal brain areas (Wiggins et al. 2016). This technique assumes a positive correlation between changes in HbO and HbR concentrations in systemic physiological signals, but a negative correlation between the two chromophores in the functional (cortical) response (Yamada et al. 2012).

The haemodynamic response amplitude was calculated on a channel-wise basis using a general linear model approach (Schroeter et al. 2004) to enable statistical analyses to be performed. The design matrix was comprised of a set of three regressors for each auditory condition and an additional set for the silent condition, corresponding with the canonical haemodynamic response (provided in SPM8 [http://www.fil.ion.ucl.ac.uk/spm]) and its first two temporal derivatives (to capture longer responses or those which had shifted in time) (Friston et al. 1998; Lindquist and Wager, 2007; Lindquist et al. 2009; Wijayasiri et al. 2017; Mushtaq et al. 2019). Each individual trial was modelled as an epoch corresponding to the duration of the stimulus for that trial. The canonical and temporal-derivative regressors were then serially orthogonalised with respect to one another for each condition (Calhoun et al. 2004). Note that an additional set of regressors-of-no interest corresponding with the reward stars were incorporated into the model to ensure that any related brain activity was captured, although not of interest. The probe word recognition stage of each trial was not explicitly modelled, on the assumption that any motor-related brain activity would cancel out due to a similar button-press response being required on every trial. Model estimation was carried out using a dual-stage least squares technique (Plichta et al. 2007), and the Cochrane and Orcutt (1949) method was applied to correct for serial correlation. Finally, overall estimated response amplitudes (ERAs) were calculated by combining the beta weights corresponding to the three regressors using the “derivative-boost” procedure (Calhoun et al. 2004; Steffener et al. 2010). Importantly, although fNIRS studies traditionally report both HbO and HbR results, the ERAs reported here are an estimate of the HbO response only due to the two chromophores becoming statistically redundant following application of the signal separation algorithm (Yamada et al. 2012), in which a linear relationship between the two chromophores is assumed. Note also that the fNIRS data analysis procedure was performed separately for each fNIRS imaging run (i.e., one complete run of the main fNIRS imaging task), and then, the ERAs were averaged across runs for participants who had performed two runs.

#### Statistical Analysis

Both behavioural and fNIRS data were analysed using a series of linear mixed models (LMMs), performed in MATLAB using functions from the Statistics and Machine Learning Toolbox. Model parameters were estimated using the restricted maximum likelihood (REML) approach. When conducting analyses of variance for the LMMs, we adopted a conservative approach by applying the Satterthwaite approximation to compute the degrees of freedom.

#### Behavioural Data

Speech perception data were analysed using a LMM that incorporated three fixed factors: “stimulus condition” (4-ch-F vs. 8-ch-F), “part” (pre- vs. post-imaging), and a “stimulus condition x part” interaction. A random intercept for “participant” was included in the model to account for between-subject variability. Only the partial intelligibility conditions were included in the model, due to scores in the zero and high intelligibility conditions being close to floor and ceiling level, respectively.

Accuracy and response time in the main fNIRS task were analysed separately using LMMs. As in earlier related studies (Binder et al. 2004; Lawrence et al. 2018), mean response time was calculated across all trials, regardless of whether a correct or incorrect response was made. Both models included fixed effects of “stimulus intelligibility” (zero vs. partial vs. high), “degradation type” (number of channels vs. AM depth), and “fNIRS run” (run 1 vs. run 2), as well as all two- and three-way interactions, and a random intercept for “participant.”

#### fNIRS Data

fNIRS data were initially analysed at a group level using channel-wise LMMs. For each channel, fixed effects of “stimulus intelligibility” (zero vs. partial vs. high), “degradation type” (number of channels vs. AM depth), and the interaction between the two were included in the model, as well as a random intercept for “participant.” The false discovery rate (FDR) correction by Benjamini and Hochberg (1995) was applied to account for multiple comparisons across channels. Following this, we conducted channel-wise polynomial trend analyses using a further set of LMMs to understand how brain activation in different areas varied with stimulus intelligibility. The approach taken was similar to that used in prior studies (Lawrence et al. 2018, 2021). For each channel, the model included the following fixed effects: “intercept” (representing overall activation in response to sound vs. silence), “linear relationship with stimulus intelligibility” (coded categorically, i.e. 0 = zero intelligibility, 1 = partial intelligibility, 2 = high intelligibility), orthorgonalised with respect to the intercept term, and “quadratic relationship with stimulus intelligibility,” orthogonalised with respect to the intercept and the linear terms. A random intercept for “participant” was also included.

For the purposes of visualisation, the results of channel-wise statistical analyses were projected as colour-coded surfaces over an image of the cortex, using cubic interpolation to derive a dense two-dimensional grid from the original sparsely located fNIRS channels. Alignment to the cortical image was based on 3D digitisations of optode locations that were subsequently registered to an atlas brain using the AtlasViewer tool (Aasted et al. 2015). Mean optode locations were computed across a separate group of twelve child volunteers of comparable age to the participants in the present study.

**Fig. 1:**
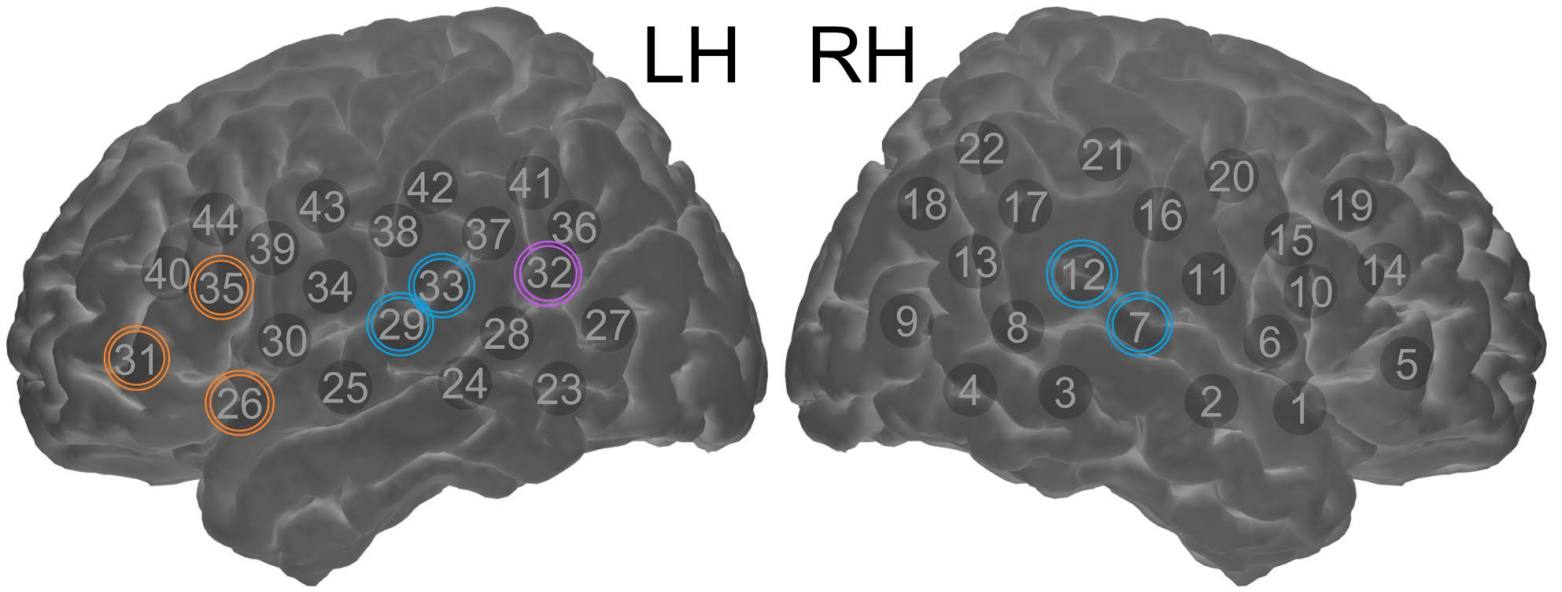
fNIRS measurement channel locations and a priori regions of interest. The channels outlined in blue form the superior temporal ROIs, which are located symmetrically in the LH and RH. The channels outlined in orange form the left inferior frontal ROI. The channel outlined in purple forms the left posterior temporal ROI

In addition to performing map-wise analyses across the optode arrays, region-of-interest (ROI) ERAs (single-subject level responses for each condition) were calculated for a priori and post hoc ROIs. The primary a priori “auditory” ROI targeted temporal brain regions and comprised symmetrical channels 29 and 33 in the left hemisphere (LH) and channels 7 and 12 in the right hemisphere (RH), the selection of which was based upon our previous fNIRS work involving auditory stimuli with children of similar age (Mushtaq et al. 2019). A pair of secondary a priori ROIs targeted “left inferior frontal” regions (channels 26, 31, and 35 in the LH) and “left posterior temporal” regions (channel 32 in the LH). Again, the selection of these ROIs was based on previous fNIRS research conducted in our laboratory with the same optode array and comparable auditory stimuli (Lawrence et al. 2018; Mushtaq et al. 2019). The data-driven post hoc ROIs were defined based on results of the channel-wise fNIRS analyses and were included to clarify the nature of the effects observed in these cortical regions. See Fig. 1 for an illustration of the position of the 44 channels and our a priori ROIs.

Finally, in order to investigate hemispheric differences, further LMMs were performed separately for a priori and post hoc ROIs targeting auditory brain regions. The models included fixed effects of “stimulus intelligibility” (treated as a continuous variable and coded as 0 = zero intelligibility, 1 = partial intelligibility, 2 = high intelligibility), “hemisphere” (left vs. right), and a “stimulus intelligibility × hemisphere” interaction. A random intercept for “participant” was also included.

## **RESULTS**

### Speech Perception

**Fig. 2:**
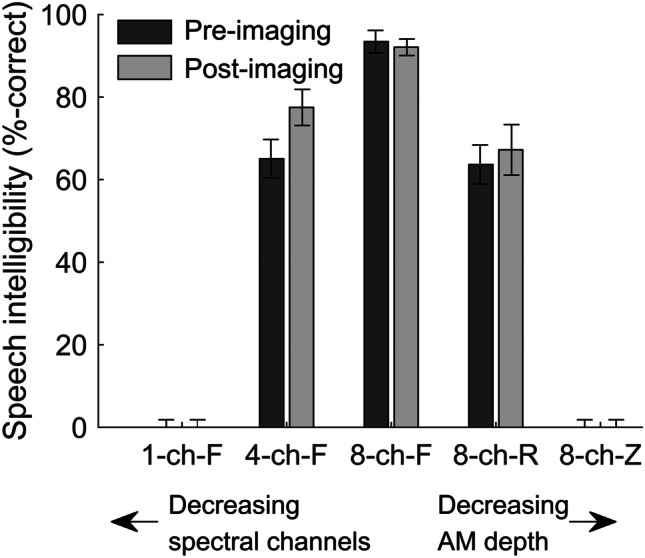
Mean speech perception scores pre- and post-imaging for the five stimulus conditions. Participants failed to identify any keywords in the two unintelligible conditions (1-ch-F and 8-ch-Z). Scores in the two partially intelligible conditions (4-chF and 8-ch-R) increased from pre- to post-imaging, suggesting a learning effect. Performance was close to ceiling level in the 8-ch-F condition. Error bars show 95 % confidence intervals corrected for a repeated-measures design following O’Brien and Cousineau (2014)

As expected, no participant correctly identified any keywords under the 1-ch-F or 8-ch-Z conditions since both stimuli were designed to be unintelligible. For the 4-ch-F condition, the group mean score was 65 % correct pre-imaging and 77 % post-imaging. For the 8-ch-R condition, group mean performance increased from 64 % correct pre-imaging to 67 % correct post-imaging. Furthermore, pre- and post-imaging averaged scores were close for these two conditions (71 % for 4-ch-F and 65 % for 8-ch-R), indicating that our aim to make these two conditions similarly challenging was largely met. For the 8-ch-F condition, scores remained consistent pre-imaging (93 %) to post-imaging (92 %). This is unsurprising given that this stimulus is considered to be highly intelligible in adult listeners (Lawrence et al. 2018); thus, learning effects are likely to be less pronounced. Speech perception scores are displayed in Fig. 2.

In order to explore whether performance differed significantly between the two partially intelligible listening conditions (4-ch-F and 8-ch-R), a LMM was performed. The main effect of stimulus condition was not statistically significant (*F*(1,84) = 0.156, *p* = 0.69). The main effect of part was significant (*F*(1,84) = 12.469, *p* < 0.001), indicating a significant improvement from pre- to post-imaging. Although the training effect appeared to have been slightly larger for 4-ch-F than for 8-ch-R, the interaction between stimulus condition and part did not reach statistical significance (*F*(1,84) = 3.180, *p* = 0.078).

### Accuracy and Mean Response Time

**Fig. 3:**
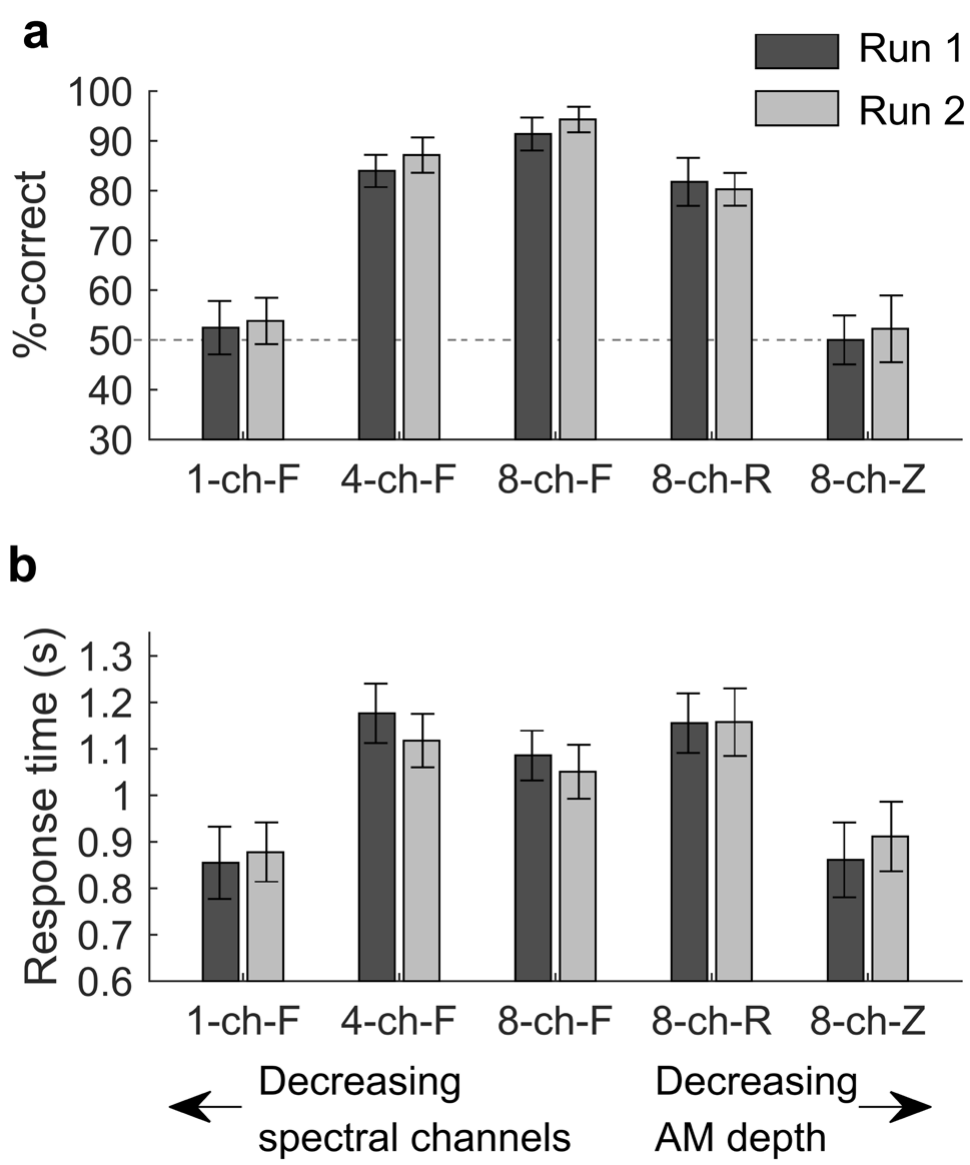
Mean accuracy and response time for the word recognition task completed during fNIRS imaging. Percentage of correct responses is shown in panel **a**, and mean response time is shown in panel **b**. For both measures, performance remained consistent across fNIRS runs but differed significantly between stimulus conditions (*p* < 0.001). Response accuracy was approximately at chance (50 %) under the two unintelligible listing conditions (1-ch-F and 8-ch-Z) but improved as stimuli became more intelligible. Response times peaked under the partially intelligible (4-ch-F and 8-ch-R) conditions and were at an intermediate level under the most intelligible condition (8-ch-F). Error bars show 95 % confidence intervals corrected for a repeated-measures design following O’Brien and Cousineau (2014)

Behavioural performance in the main fNIRS task was analysed in terms of accuracy and response time. For accuracy, there was a statistically significant main effect of stimulus intelligibility, (*F*(2,295.8) = 217.97, *p* < 0.001). There was no significant main effect of degradation type (indicating no overall difference between reducing channels vs. reducing AM depth), and no significant main effect of fNIRS run (indicating no overall training or fatigue effect between runs), as well as no significant interactions between any combination of factors (all *p* > 0.05). For response time, the statistical results were very similar: a significant main effect of stimulus intelligibility only (*F*(2,295.33) = 51.137, *p* < 0.001), with all other main effects and interactions not reaching statistical significance (all *p* > 0.05). It is worth noting that mean response time varied non-monotonically with stimulus intelligibility. Specifically, participants made their responses most quickly in the unintelligible conditions (1-ch-F and 8-ch-Z) and responses were slowest in the partially intelligible conditions (4-ch-F and 8-ch-R). Response time was intermediate for highly intelligible sentences (8-ch-F). Bar plots displaying mean accuracy scores and response times across all five stimulation conditions are displayed in Fig. 3a and b, respectively.

### Neuroimaging Results

#### Data Pre-processing

Following the fNIRS data pre-processing steps, including the exclusion of channels with poor signal quality using the SCI method and the application of motion artefact correction, a total of 9.9 % of all channels were excluded from the final analyses. Usable data were obtained from all twenty-nine participants.

#### Channel-wise Analyses

**Fig. 4:**
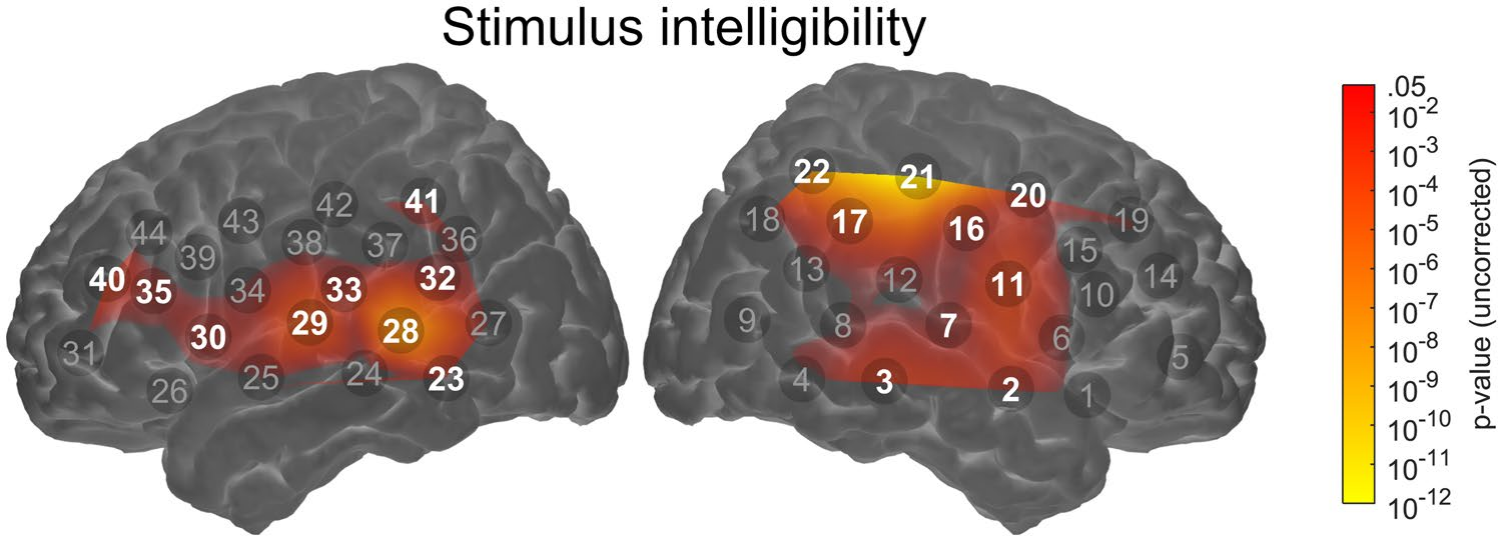
Group-level channel-wise statistical map for the main effect of stimulus intelligibility. The map is thresholded at an uncorrected *p*-value of .05. The nine channels in each hemisphere that showed a significant result after FDR correction (*q* < .05) are highlighted. Note that the map is interpolated from single-channel results and the overlay on the cortical surface is for illustrative purposes only

fNIRS data were initially analysed using channel-wise LMMs. After FDR correction, a total of nine channels in each hemisphere covering a large proportion of the optode array showed a statistically significant main effect of stimulus intelligibility (*q* < 0.05). Figure 4 shows a group-level statistical map illustrating these results. No channels showed a significant main effect of degradation type or a stimulus intelligibility x degradation type interaction (all *q* > 0.05).

**Fig. 5:**
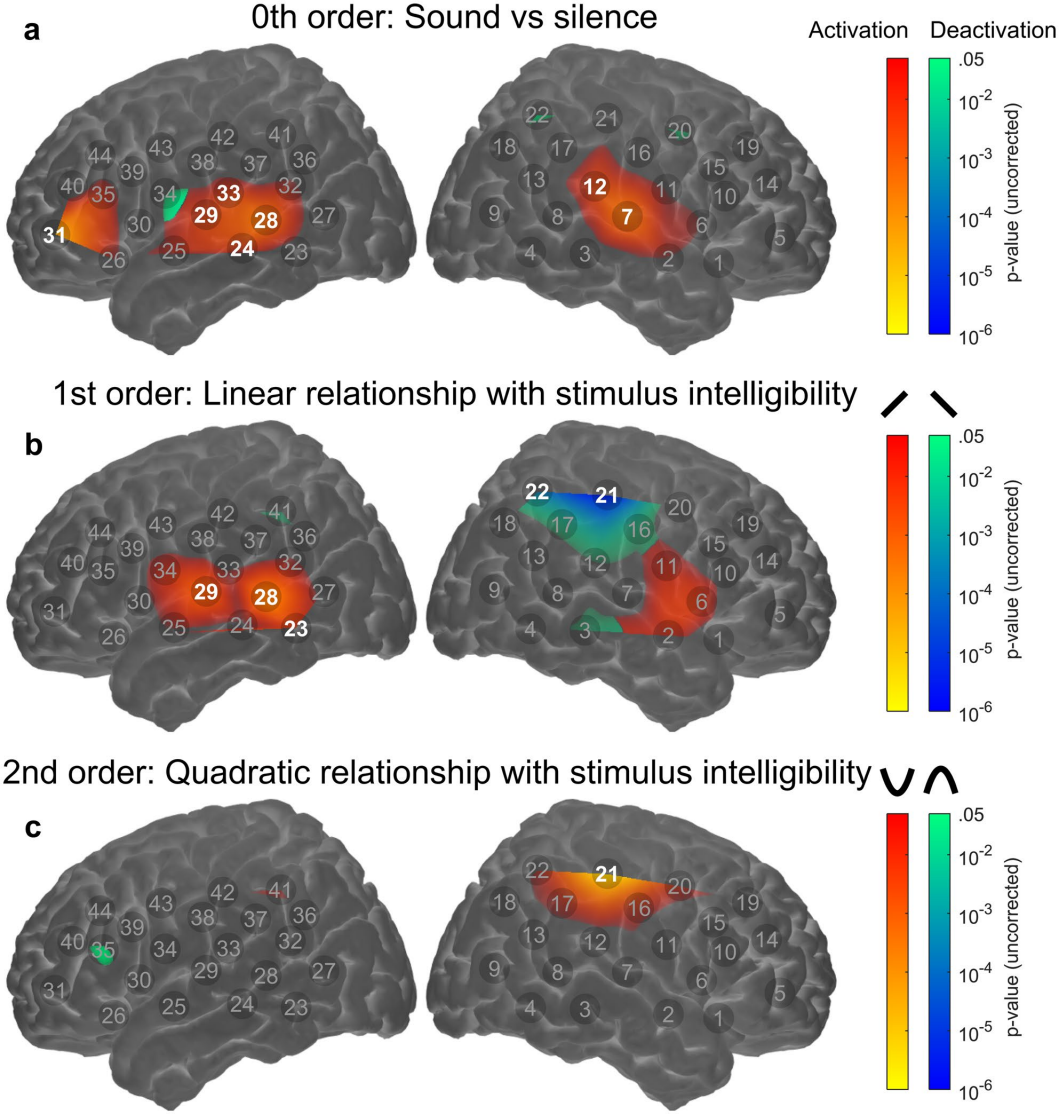
Group-level channel-wise relationships between fNIRS response amplitude and stimulus intelligibility. Rows **a**–**c** show the results of statistical significance testing (uncorrected *p*-values, thresholded at *p* < .05) for 0th-order, 1st-order (linear), and 2nd-order (quadratic) effects, respectively. Note that responses were averaged across conditions of similar stimulus intelligibility (i.e., across 1-ch-F and 8-ch-Z, and across 4-ch-F and 8-ch-R). Individual channels exhibiting significant effects after FDR correction (*q* < .05) are highlighted. Two separate colour scales are used to indicate effects in either direction, positive or negative. Note that the maps are interpolated from single-channel results and the overlay on the cortical surface is for illustrative purposes only

While the initial analysis identified channels that were significantly responsive to stimulus intelligibility, it did not provide insight into *how* response patterns changed in each of those channels as the intelligibility of the stimulus was varied. Consequently, we performed channel-wise polynomial trend analyses. Since the initial analysis did not reveal any significant main effects or interactions involving degradation type, we averaged across conditions of similar stimulus intelligibility (across 1-ch-F and 8-ch-Z, and across 4-ch-F and 8-ch-R). Figure 5a shows the 0th-order effect (i.e., overall activation or deactivation in response to sound vs. silence, regardless of intelligibility). After FDR correction, a total of 5 channels in the LH and 2 channels in the RH were significantly activated in response to sound vs. silence, (*q* < 0.05). Whilst in the RH significant activation was confined to the a priori “auditory” ROI (channels 7 and 12), in the LH, significant activation extended to more posterior temporal channels (channels 24 and 28) and to the left inferior frontal cortex (channel 31). No channels were significantly deactivated in response to sound vs. silence.

First-order effects (linear relationship with stimulus intelligibility) are displayed in Fig. 5b. LH channels overlying the a priori “auditory” ROI (channel 29) as well as more posterior temporal regions (channels 23 and 28) showed a positive linear relationship with stimulus intelligibility, indicating that more intelligible stimuli elicited stronger responses in these regions. A cluster of channels in right superior temporal/inferior frontal regions (channels 2, 6, 11) showed a similar trend, although the effect did not reach statistical significance in any individual channel after FDR correction (*q* > 0.05). In a right inferior parietal region (channels 21 and 22), there was a significant negative linear relationship between cortical responses and stimulus intelligibility (*q* < 0.05). In this same region of right inferior parietal cortex (centred on channel 21), there was also a significant quadratic component to the relationship between stimulus intelligibility and cortical responses (*q* < 0.05; see Fig. 5c). There was some evidence of a trend towards an “inverted-U” response pattern in the left inferior frontal cortex (channel 35); however, this effect did not reach statistical significance after FDR correction (*q* > 0.05).

#### fNIRS ROI Plots

**Fig. 6:**
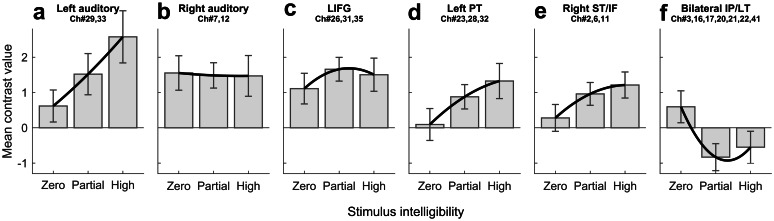
Mean contrast values (i.e., ERAs relative to silence, arbitrary units) for a priori and post hoc ROIs. Panels **a**–**c** show bar plots derived from a priori ROIs: left auditory, right auditory, and left inferior frontal gyrus (LIFG), respectively. Panels **d**–**f** show bar plots derived from post hoc ROIs: left posterior temporal (PT), right superior temporal/inferior frontal (ST/IF), and bilateral inferior parietal/lateral temporal (IP/LT). The post hoc ROIs were defined based on significant effects in the channel-wise fNIRS analyses illustrated in Fig. 5. Thick black lines show the best quadratic fit to the data. Response amplitude increased with rising stimulus intelligibility in the left, but not right, auditory ROI. Response amplitude also increased with rising stimulus intelligibility in the left PT and right ST/IF ROIs. In the LIFG, response amplitude was highest for partially intelligent speech. In bilateral IP/LT regions, a trend towards deactivation was observed, with the greatest deactivation occurring for partially intelligible speech. Error bars show 95 % confidence intervals corrected for a repeated-measures design following O’Brien and Cousineau (2014)

To help visualise the response patterns in specific cortical regions, we produced bar plots of fNIRS ERAs (see Fig. 6) for each of several ROIs. The ROIs comprised a mixture of a priori (left and right “auditory” ROIs and “left inferior frontal gyrus” ROI; Fig. 6a, b, and c, respectively) and post hoc channel groupings (“left posterior temporal,” “right superior temporal/inferior frontal,” and “bilateral inferior parietal/lateral temporal” ROIs; Fig. 6d, e, and f, respectively). Figure 6b confirms that responses in the a priori right “auditory” ROI were largely independent of stimulus intelligibility. There is again a suggestion that left inferior frontal gyrus responses (Fig. 6c) were strongest to partially intelligible stimuli, but this was a relatively weak effect that did not reach statistical significance in any individual fNIRS channel. In bilateral inferior parietal/lateral temporal regions, the response to sentences was generally a relative deactivation, with the strength of the deactivation being greatest for partially intelligible speech.

**Fig. 7:**
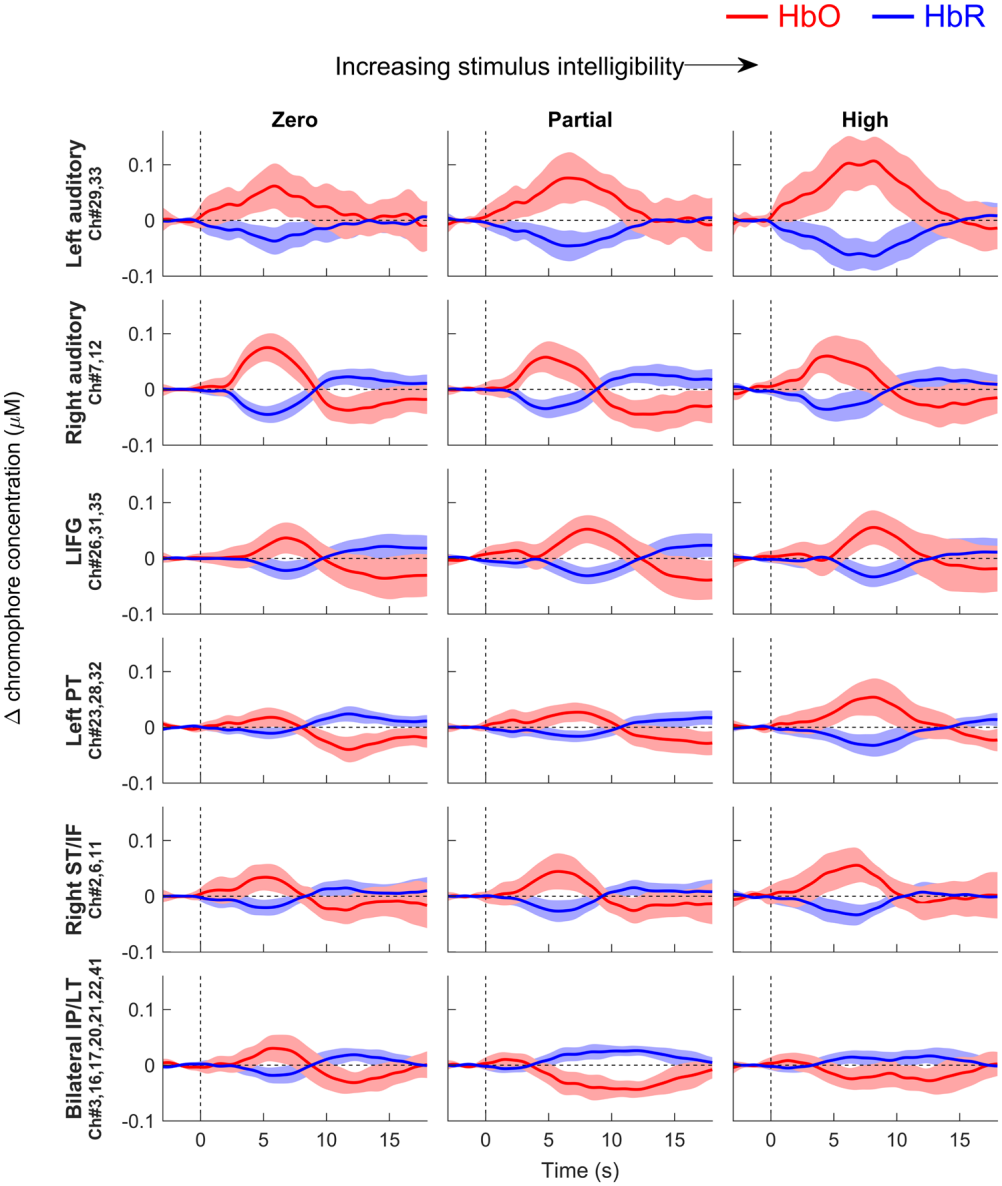
Event-averaged haemodynamic time courses for a priori and post hoc ROIs. The red and blue traces show estimated changes in the concentration of HbO and HbR, respectively (average response to silent trials subtracted out). The shaded area represents the 95 % confidence interval around the group mean. The first three rows show time courses for a priori ROIs: left auditory, right auditory, and left inferior frontal gyrus (LIFG), respectively. The lowermost three rows show time courses for post hoc ROIs: left posterior temporal (PT), right superior temporal/inferior frontal (ST/IF), and bilateral inferior parietal/lateral temporal (IP/LT)

Event-averaged haemodynamic time courses were also plotted for the same ROIs (see Fig. 7). Interestingly, LH auditory responses appeared to have been more elongated in time than RH auditory responses. As in previous studies (e.g., Wijayasiri et al. 2017), responses in the left inferior frontal gyrus appear slightly delayed relative to responses in auditory regions. Furthermore, the relative deactivation observed in bilateral inferior parietal/lateral temporal regions appeared to be particularly diffuse and elongated in time.

#### Hemispheric Differences

**Fig. 8:**
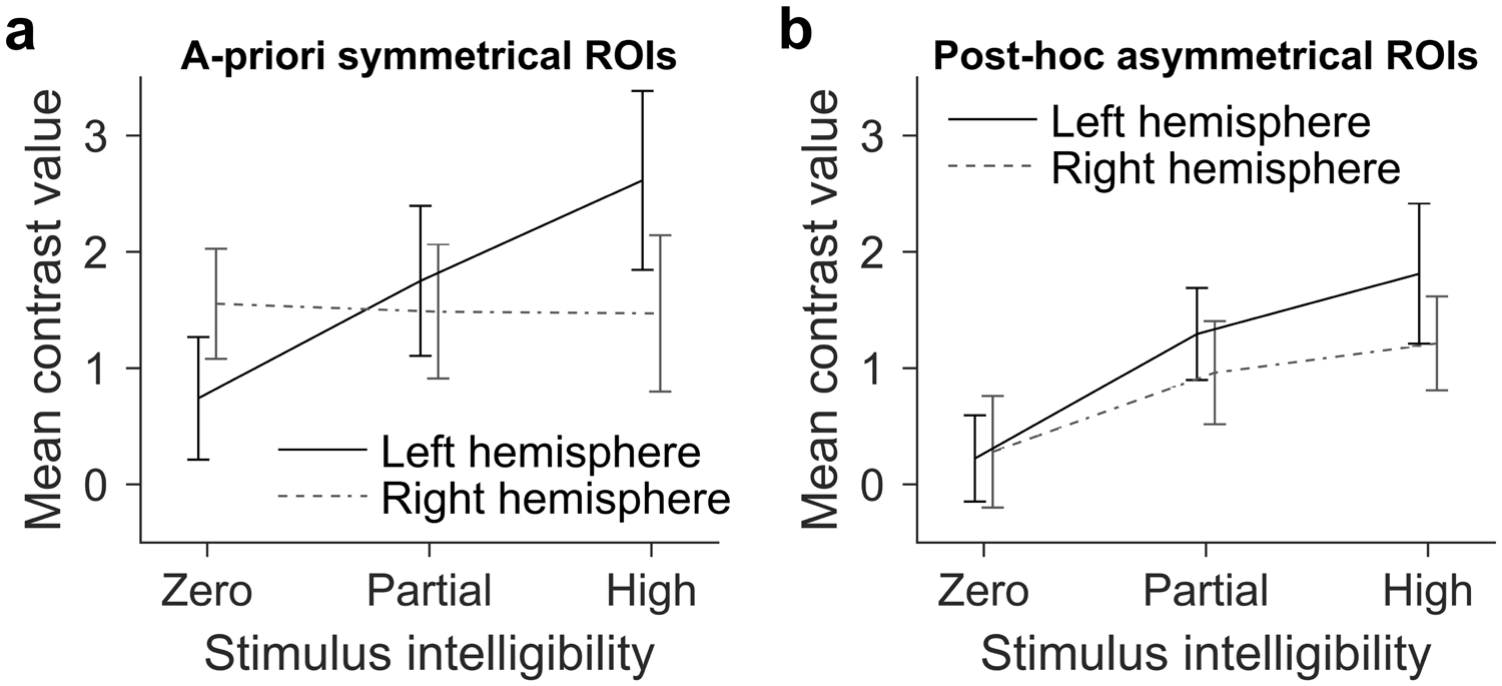
Mean contrast values (i.e., ERAs relative to silence, arbitrary units) as a function of stimulus intelligibility and hemisphere in a priori and post hoc ROIs. Panel **a** shows results for the a priori auditory ROIs (channels 29 and 33 in the LH and channels 7 and 12 in the RH). Response amplitude increases with rising stimulus intelligibility, but only in the LH, as confirmed by a significant stimulus intelligibility × hemisphere interaction (*p* < 0.001). Panel **b** shows results for the post hoc ROIs, which are the channels that showed the strongest evidence of a positive linear relationship with stimulus intelligibility (asymmetrical channels 23, 28, and 29 in the LH and channels 2, 6, and 11 in the RH). In these ROIs, responses in the LH and RH showed a more similar pattern, increasing as stimuli became more intelligible. Error bars show 95 % confidence intervals corrected for a repeated-measures design following O’Brien and Cousineau (2014)

In the a priori (symmetrical) “auditory” ROIs, our analyses indicated that a relationship between fNIRS response amplitude and stimulus intelligibility existed in the LH, but not in the RH (see Fig. 6a and b). However, to conclude that the relationship is truly unique to the LH, a statistically significant stimulus intelligibility × hemisphere interaction is required. The presence of this interaction was confirmed using a LMM (*F*(1,138.67) = 11.513, *p* < 0.001; see Fig. 8a).

Since there were other channels in the RH, outside of the a priori “auditory” ROI, that appeared to show some evidence of a positive relationship with stimulus intelligibility, we also compared hemispheric differences in fNIRS response amplitude in a second, post hoc ROI, based on channels that showed the strongest evidence for a positive linear relationship with stimulus intelligibility in each hemisphere in Fig. 5b (channels 23, 28, and 29 in the LH and channels 2, 6, and 11 in the RH), regardless of whether they were symmetrically located. The results are displayed in Fig. 8b. Using these post hoc ROIs, the difference between the LH and RH was less pronounced, and the corresponding LMM showed the stimulus intelligibility × hemisphere interaction to no longer reach statistical significance (*F*(1,139.12) = 3.2834, *p* = 0.072).

### Effects of Age

**Fig. 9:**
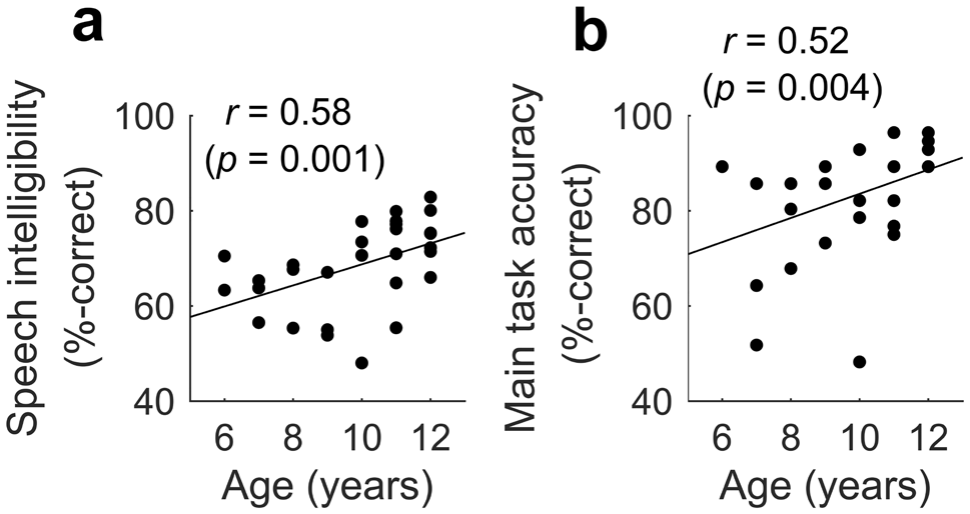
Scatter plots of behavioural performance against participant age. Positive correlations were observed between speech intelligibility and age (panel **a**) and between accuracy on the word recognition task conducted during fNIRS imaging and age (panel **b**)

Although age effects were not of direct interest in this study, because our sample included children whose ages spanned a range of 6 years, we tested for the presence of any relationship between age and (i) speech intelligibility and (ii) accuracy on the main fNIRS imaging task (see Fig. 9a and b, respectively, for scatter plots of the data). Spearman’s rho correlations (which were deemed appropriate due to the spread of the data points) revealed a significant positive correlation between age and speech intelligibility (*r*_*s*_ = 0.58, *p* = 0.001), as well as age and main task accuracy (*r*_*s*_ = 0.52, *p* = 0.004). Consequently, all of the fNIRS analyses reported above were re-run with age included as an additional fixed effect in the LMMs. In all cases, the results of the original analyses were unaffected, with no significant effects of age observed. This suggests that the patterns of brain activity observed in the present study were not heavily influenced by chronological age in the range 6 to 12 years.

## **DISCUSSION**

We presented NH children with noise-vocoded speech to study the impact of varying speech intelligibility on functional responses recorded from both cerebral hemispheres using fNIRS.

In our initial channel-wise analysis, 18 channels covering a large proportion of the optode array showed a significant effect of stimulus intelligibility, highlighting the complex and distributed nature of cortical speech processing. Interestingly, in the LH, these channels were largely confined to regions known to play an important role in language processing (Lazard et al. 2012), while in the RH, the location of the channels showing sensitivity to speech intelligibility was more diffuse.

Our analyses revealed a positive linear relationship between stimulus intelligibility and channels targeting auditory regions (differing slightly from our a priori ROI although with some overlap). Our data suggested that this positive relationship was stronger in the LH than in the RH, although there was weak evidence of a similar response profile in RH channels located outside of our a priori ROI. An effect of stimulus intelligibility confined to the LH might be expected given the dominant role of the LH in speech processing (Lazard et al. 2012). However, previous fNIRS studies with adult participants have revealed more symmetrical response patterns to changes in speech intelligibility covering both hemispheres (Pollonini et al. 2014; Lawrence et al. 2018). Further research is needed to establish whether the predominantly left-lateralised relationship with speech intelligibility observed in the present study reflects a true difference between cortical speech processing in children versus adults, or whether this finding is better explained by other factors such as differences in stimuli between studies, the limited spatial resolution of fNIRS, or even just statistical chance. While we did not detect any significant influence of age on cortical measurements within our sample of children aged 6–12 years, differences in the lateralisation of speech processing between children and adults are certainly plausible, given that auditory and language areas of the brain are known to continue to develop well into adolescence (Giedd et al. 1999; Lenroot and Giedd, 2006). Additionally, our fairly wide paediatric age group may have masked more complex lateralisation effects, such as changing lateralisation patterns (e.g., an age-related reduction in RH activation) between childhood and adulthood, for example, as shown in a recent fMRI language study conducted by Olulade et al. (2020).

We expected the two partially intelligible conditions (4-ch-F and 8-ch-R) to elicit the greatest listening effort in our participants, on the basis that speech understanding under these conditions would be possible, but highly challenging. The mean response time data were consistent with this notion, in that it took participants around 7 % longer to make their responses in the 4-ch-F and 8-ch-R conditions compared to the more intelligible 8-ch-F condition. Mean response times were markedly shorter for unintelligible stimuli (1-ch-F and 8-ch-Z conditions), suggesting that most participants identified these trials as being impossible and simply guessed at the correct response. Based on prior fNIRS and fMRI findings (Wild et al. 2012; Wijayasiri et al. 2017; Lawrence et al. 2018), we hypothesised that greater listening effort in the partially intelligible conditions would be associated with maximal activation in the left inferior frontal cortex. Our data showed a clear trend in this direction (“inverted-U” response profile across stimulus intelligibility levels), however the effect did not reach statistical significance in any individual fNIRS channel after correcting for multiple comparisons. Prior studies have also revealed a consistent trend towards relative deactivation of inferior parietal and/or lateral temporal areas during a challenging speech understanding task, with the strength of the deactivation being greater under more effortful conditions (Wild et al. 2012; Lawrence et al. 2018). The same pattern was clearly evident in the present dataset, with the effects being particularly pronounced in the RH. We suggest that this difficulty-dependent deactivation likely reflects reduced activity in the default-mode network during effortful listening (Buckner et al. 2008; Lawrence et al. 2018, 2021). It is possible that we might have observed stronger evidence of listening-effort-related brain activity, particularly in the left inferior frontal cortex, had we included a clear (un-vocoded) reference condition: this would have presented a greater contrast to the partially intelligible conditions, since the 8-ch-F condition, while highly intelligible, may in itself have required significant investment of listening effort to understand. A clearer interpretation of these data might have been facilitated by the simultaneous measurement of listening effort using a complementary technique, such as pupillometry or a dual-task paradigm (McGarrigle et al. 2014).

We hypothesised that stronger responses would be observed in our left posterior ROI (channel 32) in response to more intelligible stimuli, reflecting higher-level speech processing (Wise et al. 2001). While we did not identify a statistically significant linear relationship with stimulus intelligibility in this channel (after correcting for multiple comparisons), we did observe this pattern in neighbouring channels 28 and 23. It seems likely that our a priori selection of channel 32 as a ROI was too restrictive to capture the relevant cortical activity, given the limited spatial resolution of fNIRS (Quaresima et al. 2012). The fact that we did not observe a significant main effect of degradation type or a significant intelligibility x degradation type interaction in any fNIRS channel, despite widespread and strongly significant main effects of intelligibility, suggests that the brain activity picked up by our fNIRS measurements was largely insensitive to the specific acoustic form of the stimuli, but rather just to the ease with which the speech could be understood. That said, it is important to acknowledge that our analyses do not show relationships with “intelligibility” per se, but rather with categories of “stimulus intelligibility” (i.e., zero, partial and high), so the present findings must be interpreted with this caveat in mind.

Another limitation worthy of consideration is the unknown effect of reading ability on our results, since children at the lower vs. upper age limit will have differed in their reading ability and the number of words known to them (Kail and Hall, 1994; Rice and Hoffman, 2015). However, BKB sentences (the auditory stimuli used throughout all of the tasks) are simple, contain common words, and are often used to measure behavioural speech perception in school-age children in the UK, including patient groups (Bench et al. 1979). Furthermore, when analyses were re-run with age included as a factor, the results remained unchanged. Of course, whilst absence of evidence should not be taken as evidence of absence, these findings would appear consistent with the idea that although behavioural performance improved with age, there does not seem to have been any fundamental difference in how these stimuli were processed within the brains of children of different ages within our sample.

Finally, it is important to acknowledge that controversy surrounds the use of vocoders to simulate, in NH listeners, the experience of degraded listening in clinical populations (such as cochlear implant recipients, for example). This is because the former group listens to modified signals through a normal auditory periphery, while the latter group listens to normal signals (cochlear implant sound processing not withstanding) through a highly modified auditory periphery. The application of findings from NH listeners to patient groups therefore has to be treated with caution. Nonetheless, there is evidence to suggest that cortical response patterns to auditory (including vocoded) stimuli are similar between NH listeners and some clinical populations, at least in adults (Olds et al. 2016). Therefore, there is cause for optimism that the findings of vocoder studies with NH individuals can at least provide useful clues as to how auditory processing and speech perception might occur within the brains of cochlear implant recipients, for example.

In conclusion, our noise-vocoded stimuli elicited statistically significant cortical responses, measured using fNIRS in NH children. Auditory regions in the LH were responsive to changes in stimulus intelligibility, indicating that cortical differences in speech perception can be revealed in these areas. The use of vocoded stimuli offers a valuable insight into auditory processing in NH children, and findings could be used to inform the future development of tools for measuring, monitoring and improving speech perception in paediatric populations.
